# The Effect of C-MAC Miller 1 Blade Placement in the Vallecula Versus Direct Epiglottic Elevation on Glottic Visualization and Intubation Performance in Children Under 2 Years of Age: A Prospective Randomized Controlled Trial

**DOI:** 10.3390/jcm15197483

**Published:** 2026-09-26

**Authors:** Ahmet Kenç, Zekine Begeç, Ülkü Özgül, Cemil Çolak, Oya Olcay Özdeş

**Affiliations:** 1 Department of Anesthesiology and Reanimation, Battalgazi State Hospital, Malatya 44210, Turkey; akenc44@gmail.com; 2 Department of Anesthesiology and Reanimation, Faculty of Medicine, İnönü University, Malatya 44210, Turkey; zekine.begec@inonu.edu.tr (Z.B.); ulku.ozgul@inonu.edu.tr (Ü.Ö.); 3 Department of Biostatistics, and Medical Informatics, Faculty of Medicine, İnönü University, Malatya 44210, Turkey; cemil.colak@inonu.edu.tr

**Keywords:** airway management, anesthesia, pediatric, video laryngoscope

## Abstract

**Background/Objectives**: This study aimed to compare glottic visualization and intubation performance during laryngoscopy with the C-MAC video laryngoscope size 1 Miller blade using direct epiglottic elevation versus vallecular placement of the blade tip in children younger than 2 years of age. **Methods**: This prospective, randomized controlled trial enrolled 127 ASA physical status I–II children younger than 2 years undergoing elective surgery. Participants were randomly allocated to Group V (vallecular placement; n = 62) or Group E (direct epiglottic elevation; n = 65). Anesthesia was induced with fentanyl, thiopental, sevoflurane, and rocuronium. The Percentage of Glottic Opening (POGO) score, Cormack–Lehane grade, Intubation Difficulty Score (IDS), Visual Analogue Scale (VAS) score, laryngoscopy and intubation times, intubation attempts, optimization maneuvers, and complications were recorded. **Results**: The POGO score was significantly higher in Group E (median [IQR], 96 [92–100]) than in Group V (95 [88–100]; *p* = 0.032). Cormack–Lehane grades were significantly better in Group E than in Group V (*p* = 0.042). Laryngoscopy time was significantly shorter in Group V (5.61 ± 1.15 s) than in Group E (6.95 ± 1.80 s; *p* = 0.001). Intubation time was also significantly shorter in Group V (23.16 ± 4.44 s) than in Group E (26.60 ± 4.73 s; *p* = 0.001). VAS scores were significantly higher in Group V (9.48 ± 1.09) than in Group E (9.02 ± 1.09; *p* = 0.018). **Conclusions**: Direct epiglottic elevation with the C-MAC video laryngoscope size 1 Miller blade improved glottic visualization, whereas vallecular placement was associated with shorter laryngoscopy and intubation times in children younger than 2 years. The choice between the two techniques may depend on clinical priorities; vallecular placement may be considered when minimizing intubation time is a priority, although this hypothesis requires prospective evaluation in higher-risk populations.

## 1. Introduction

The more cephalad location of the larynx and its proximity to the tongue base in neonates and infants may complicate the visualization of the laryngeal structures and make tracheal intubation more challenging. In this age group, airway management is also physiologically demanding because of limited oxygen reserves in addition to these anatomical differences, substantially reducing the time available for securing the airway [1,2].

Direct laryngoscopy (DL) remains a widely used method for airway management. The Miller laryngoscope blade is commonly used in pediatric patients younger than 2 years because its straight design allows direct elevation in the relatively long and floppy epiglottis, thereby facilitating visualization of the laryngeal inlet [3]. In the classical technique, the tip of the Miller blade is advanced beneath the epiglottis, producing direct mechanical elevation in the epiglottis. Alternatively, the blade tip can be positioned in the vallecula, similar to the Macintosh technique, resulting in indirect elevation in the epiglottis [4]. Thus, the two blade positions differ biomechanically in the mechanism by which the epiglottis is elevated, which may influence both glottic visualization and the time required for laryngoscopy and intubation. A previous study in children younger than 2 years found no difference in optimal laryngeal visualization between direct elevation in the epiglottis with a Miller 1 blade and placement of the blade tip in the vallecula [5].

Video laryngoscopes (VLs) have increasingly been incorporated into clinical airway management. Whereas the viewing angle during DL is constrained by the oropharyngeal structures, VL provides a wider viewing angle and an enlarged image of the laryngeal structures [6]. A Miller blade specifically designed for pediatric tracheal intubation is available for the C-MAC VL system (Karl Storz, Tuttlingen, Germany), and video laryngoscopy is increasingly used for endotracheal intubation in pediatric patients [7,8,9].

To our knowledge, no previous study has directly compared direct epiglottic elevation with vallecular blade-tip placement using the C-MAC VL No. 1 Miller blade. We hypothesized that direct epiglottic elevation with the C-MAC VL No. 1 Miller blade would provide superior glottic visualization compared to vallecular placement, potentially at the cost of increased intubation time. Therefore, this study aimed to compare glottic visualization and intubation performance between these two blade positions in children younger than 2 years undergoing elective surgery with normal airways.

## 2. Materials and Methods

### 2.1. Study Protocol

This study was approved by the İnönü University Institutional Review Board (IRB 2021/153; approval date: 18 May 2022) in accordance with the Declaration of Helsinki, and written informed consent was obtained from the parents of all participating children. The trial was prospectively registered at ClinicalTrials.gov (NCT05515107; registration date: 25 August 2022) before enrollment of the first participant on 1 September 2022. Participant recruitment was conducted from 1 September 2022 to 30 September 2022, and study data collection was completed on 10 October 2022. The preregistered primary outcome was the Percentage of Glottic Opening (POGO) score, and the preregistered secondary outcome was intubation time. Additional outcomes reported in this manuscript, including Cormack–Lehane grade, Intubation Difficulty Score (IDS), Visual Analogue Scale (VAS) score, laryngoscopy time, number of intubation attempts, optimization maneuvers, and complications, were prospectively collected according to the study protocol but were not preregistered and are therefore considered exploratory. The study was conducted in accordance with Good Clinical Practice guidelines and the applicable Consolidated Standards of Reporting Trials (CONSORT) guidelines; the CONSORT checklist is provided in the Supplementary Materials.

### 2.2. Inclusion Criteria of the Study

Children younger than 2 years of age who were classified as ASA physical status I or II and who were scheduled to undergo elective surgery under general anesthesia requiring tracheal intubation at İnönü University Faculty of Medicine were eligible for inclusion in the study.

### 2.3. Exclusion Criteria of the Study

The patients who had an upper respiratory tract infection (URTI) within four weeks before surgery, patients who were expected to have a difficult airway in the preoperative evaluation, and who were unstable during intubation because of underlying respiratory or cardiovascular problems were not included in the study.

### 2.4. Study Design, Randomization and Blinding

The study was designed as a prospective, randomized, parallel-group clinical trial. Of the 130 children assessed for eligibility, three were excluded before randomization because of an upper respiratory tract infection within the preceding four weeks, leaving 127 eligible participants for randomization. The randomization sequence was generated by a statistician (C.Ç.) using the MedCalc statistical software package (version 20; www.medcalc.org) with a simple randomization method. Allocation concealment was ensured using sequentially numbered, opaque, sealed envelopes prepared by C.Ç., who had no clinical involvement in the study. Patient enrollment was performed by the anesthesiologist (A.K.), who opened the envelope assigned to each participant immediately before anesthesia induction.

Children in whom the C-MAC Miller blade tip was placed in the vallecula were allocated to Group V (n = 62), whereas those in whom the blade tip was placed under the epiglottis for direct epiglottic elevation were allocated to Group E (n = 65). All 127 randomized participants received their allocated intervention and were analyzed in their originally assigned groups.

Because the blade position was visible to the intubating anesthesiologist, blinding of the operator was not feasible. All intubation times were recorded by an independent observer who was unaware of group allocation. For POGO assessment, the video recordings were labelled using anonymized study codes without patient identifiers or explicit group labels and were evaluated at the end of the study by an independent anesthesiologist (Ü.Ö.) who had not participated in the intubation procedures and was unaware of the assigned group. However, because the blade tip and its relationship to the epiglottis could potentially be visible in the video recordings, complete blinding of the POGO assessor to the intervention could not be guaranteed, and inadvertent inference of group allocation was therefore possible.

### 2.5. Anesthesia Management

The characteristics such as age, gender, body weight, height, and ASA classifications of the patients were recorded before they were admitted to the surgery room. Premedication was not applied to any of the cases. Intraoperative monitoring was achieved with ECG, non-invasive blood pressure, capnography, and pulse oximetry. The shoulders of all subjects were slightly elevated with a towel roll during mask ventilation. After three minutes of preoxygenation, fentanyl (1 mcg/kg IV), thiopental (5 mg/kg IV), and inhaled sevoflurane (5–8%) with oxygen were used for anesthesia induction. Rocuronium (0.6 mg/kg IV) was administered for muscle relaxation to facilitate tracheal intubation. Muscle relaxation was confirmed by using a neuromuscular monitor. Endotracheal intubations (ETIs) were performed with the Storz C-MAC VL No. 1 Miller blade (Karl Storz, Tuttlingen, Germany) by approaching paraglossally (by entering the blade from the right commissure of the mouth opening and pushing the tongue to the left inside the mouth). In all cases, uncuffed reinforced endotracheal tubes (ETTs) of age-appropriate size with stylets were used. Glottic visualization was recorded prior to any laryngeal manipulation during video laryngoscopy and saved to the external memory card of the C-MAC VL for subsequent blinded evaluation. Cormack–Lehane (CL), Intubation Difficulty Score (IDS), and subjective intubation difficulty scores were recorded.

All intubation attempts were performed by a single pediatric anesthetist who had performed more than 50 ETIs in children with the C-MAC Miller VL to avoid variations. Unsuccessful intubation was defined as an intubation attempt that could not be achieved despite 2 attempts, or in which the total intubation time was over 60 s.

The primary outcome variable in the study was the POGO score, which corresponds to the percentage of patency of the glottis. Complete visualization of the vocal cords of the glottis from the anterior commissure to between the arytenoid notches means that the POGO score is 100%, and if the glottic opening is not visible at all, the POGO score is taken at 0%. At the end of the study, the POGO score was evaluated by an independent anesthesiologist (Ü.Ö.) who had not participated in the intubation procedures and who reviewed the anonymized video recordings without knowledge of group allocation, as described in Section 2.4. Because the position of the blade tip and its relationship to the epiglottis could potentially be inferred from the recordings, complete blinding of the POGO assessor to the intervention could not be guaranteed.

The preregistered secondary outcome was intubation time. Additional prospectively collected outcomes included the Cormack–Lehane grade, Intubation Difficulty Score (IDS), subjective ease of intubation assessed using the Visual Analogue Scale (VAS), laryngoscopy time, number of intubation attempts, optimization maneuvers performed during tracheal intubation, and intubation-related complications. As these additional outcomes were not preregistered, they were considered exploratory.

IDS is a scoring system used to measure the difficulty of intubation consisting of seven parameters that evaluate and score the ETT’s advancement towards the glottis, the number of additional operators trying ETI, the number of alternative intubation techniques used, the Cormack–Lehane Score, the lifting power applied to the laryngoscope, external laryngeal pressure, and the position of the vocal cords. The total IDS is between 0 and ∞, indicating that intubation becomes more difficult as the score increases, and a score of ∞ indicates impossible intubation. The intubator subjectively graded the ease of intubation with Visual Analogue Scale (VAS), ranging from 0 (most difficult) to 10 (easiest).

Laryngoscopy time was recorded as the time when the C-MAC VL entered the oral cavity to the time when the best laryngoscopic image recorded for the POGO score was obtained.

Intubation time was defined as the interval from insertion of the C-MAC video laryngoscope into the oral cavity until the first appearance of an EtCO_2_ trace on the capnograph. It should be noted that this operational definition differed from the prospectively registered definition, which specified measurement until two meaningful end-tidal CO_2_ readings were obtained. This deviation from the registered outcome definition is acknowledged; the operational definition was applied consistently to all participants throughout data collection. A dated protocol version predating enrollment is not available for submission as supplementary material. If the first intubation attempt failed, the duration of each attempt was recorded separately and the durations were summed, with a maximum of two attempts permitted. If the cumulative intubation time exceeded 60 s, the intubation was considered unsuccessful.

Optimization maneuvers applied during intubation were recorded as external laryngeal compression (Backward, upward and rightward pressure (BURP)), rotation of the head, and increasing the vertical lifting power.

Complications such as arterial oxygen desaturation (SpO_2_ ˂ 90%), hypertension, arrhythmia, esophageal intubation, and dental and mucosal damage were recorded during ETT placement.

### 2.6. Sample Size

The sample size was calculated a priori for the primary outcome (POGO score). The calculation was informed by previously published pediatric POGO data reported by Passi et al. [5]. Based on these data, a mean between-group difference in POGO score of approximately 5 percentage points with a pooled standard deviation of approximately 8.2 was assumed, yielding a standardized effect size of 0.61. With a two-sided Type I error (α) of 0.05 and 90% statistical power (β = 0.10), the minimum required sample size was calculated as 118 participants (59 per group) using G*Power software (version 3.1.9.7; Heinrich-Heine-Universität Düsseldorf, Düsseldorf, Germany). The calculated minimum sample size was exceeded, with 127 eligible participants ultimately randomized and included in the analysis.

### 2.7. Statistical Analysis

Quantitative data were summarized as mean ± standard deviation (SD) or as median with interquartile range (IQR), and categorical data were summarized as numbers and percentages. The distribution of quantitative variables was assessed using the Kolmogorov–Smirnov test, and homogeneity of variances was evaluated using Levene’s test. Student’s *t*-test or the Mann–Whitney U test was used for between-group comparisons of continuous variables, as appropriate according to the distributional assumptions. Categorical variables were compared using Pearson’s chi-square test, Yates’ corrected chi-square test, or Fisher’s exact test, as appropriate.

For the primary outcome (POGO score), the Kolmogorov–Smirnov test indicated a non-normal distribution in both groups (Group V: *p* < 0.001; Group E: *p* < 0.001), consistent with a pronounced ceiling effect, and Levene’s test indicated unequal variances between the groups (*p* < 0.001). Accordingly, the Mann–Whitney U test was used as the primary test for the between-group comparison of the POGO score, and the results are presented as medians with IQRs. As a distribution-appropriate effect estimate, the Hodges–Lehmann estimator of the median difference (Group E − Group V) with its 95% confidence interval is reported. As a sensitivity analysis, the parametric Student’s *t*-test was also applied, and the between-group effect estimate is reported as the mean difference (Group E − Group V) with its 95% confidence interval. All statistical tests were two-sided, and a *p* value < 0.05 was considered statistically significant. Statistical analyses were performed using IBM SPSS Statistics for Windows, version 28.0.

## 3. Results

A total of 127 participants underwent randomization, with 62 allocated to Group V and 65 to Group E. All randomized participants received the allocated intervention and were analyzed in their originally assigned groups, with no post-randomization exclusions or losses to follow-up (Figure 1). The demographic data of the groups were statistically similar (Table 1).

The intubation characteristics of the groups are shown in Table 2. The POGO score was significantly higher in Group E (median [IQR], 96 [92–100]) than in Group V (95 [88–100]; Mann–Whitney U test, *p* = 0.032). The Hodges–Lehmann estimate of the median difference (Group E − Group V) was 2.0 percentage points (95% CI, 0.5–4.0). As a sensitivity analysis, the mean POGO score was 94.80 ± 5.32 in Group E and 90.31 ± 15.83 in Group V, with a mean between-group difference of 4.49 percentage points (95% CI, 0.43–8.55; *p* = 0.032). The Cormack–Lehane score distribution (I/II/III/IV) was 54/6/2/0 in Group V and 64/1/0/0 in Group E, with a statistically significant difference (*p* = 0.042). Grade I visualization was more frequently observed in Group E (98.5%) than in Group V (87.1%). The laryngoscopy time was 5.61 ± 1.15 s in Group V and 6.95 ± 1.80 s in Group E, and the difference between the groups was statistically significant (mean difference, 1.34 s; 95% CI, 0.81–1.87; *p* < 0.001). The intubation time was 23.16 ± 4.44 s in Group V and 26.60 ± 4.73 s in Group E, and there was a statistically significant difference between the groups (mean difference, 3.44 s; 95% CI, 1.83–5.05; *p* < 0.001). The VAS value was 9.48 ± 1.09 in Group V and 9.02 ± 1.09 in Group E, and there was a statistically significant difference between the groups (mean difference, −0.46; 95% CI, −0.84 to −0.08; *p* = 0.018). The groups were statistically similar in terms of total IDS (Group V: 0.59 ± 1.24 vs. Group E: 0.49 ± 0.75; *p* = 0.567). The number of intubation attempts was similar between the groups (Group V: 61 first-attempt/1 s-attempt; Group E: 65/0; *p* = 0.488). No complications (arterial oxygen desaturation SpO_2_ < 90%, hypertension, arrhythmia, esophageal intubation, dental or mucosal damage) occurred in either group.

The optimization maneuvers in the groups shown in Table 3 and the necessary optimization maneuvers BURP were statistically similar in terms of rotation and lifting (*p* > 0.05). Only one case was intubated on the second attempt in Group V.

## 4. Discussion

In this prospective, randomized, parallel study that was conducted by using the C-MAC VL No. 1 Miller blade in children under two years of age, we found that direct epiglottic elevation with the blade tip improved the POGO score and Cormack–Lehane grade and vallecular placement of the blade tip shortened laryngoscopy and intubation times.

The C-MAC Miller VL has a straight blade and can be used as a conventional Miller laryngoscope. In other words, this device allows the insertion of the blade tip into the vallecula or direct elevation in the epiglottis during laryngeal imaging. In the literature, the C-MAC Miller VL has been used in both ways with different success rates, but no study was found comparing blade tip insertion into the vallecula or direct elevation in the epiglottis [8].

Passi et al. [5] compared direct epiglottic elevation with vallecular placement using a conventional No. 1 Miller blade during direct laryngoscopy and reported similar POGO scores between the two techniques. In contrast, the present study evaluated the same blade-positioning strategies using the C-MAC VL No. 1 Miller blade and found higher POGO scores with direct epiglottic elevation. This distinction is important because findings obtained with conventional direct laryngoscopy may not be directly transferable to video laryngoscopy. A plausible explanation is that the camera positioned at the distal end of the VL provides a closer, more direct view of the glottis when the epiglottis is elevated, thereby reducing the visual interference of surrounding anatomical structures; however, this mechanism was not directly tested in the current study.

In a study by Oh et al. [10] that investigated laryngeal visualization with VL with curved blades of different brands in adults, both POGO and CL scores were found to be better in the group in which the epiglottis was directly elevated. It has been proposed that vallecular placement of the VL blade may be suboptimal in some patients because the epiglottis can be influenced by its relationship with associated structures such as the hyoid bone and the hyo epiglottic ligament, as well as by individual anatomical variation in epiglottis size, shape, and rigidity; these remain hypothetical explanatory mechanisms and were not directly tested in the cited study or in the present trial. VLs are devices with a video camera placed at the distal end of the laryngoscope blade, which allows the user to visualize the larynx, vocal cords, and glottic opening on a video screen. In the present study, in which the Miller blade VL 1 was used in children under two years of age, both POGO and CL scores were better in the group in which the epiglottis was directly elevated, similar to the findings of Oh et al. [10].

Intubation difficulty is most evident in children younger than 2 years of age because of the anatomical and physiological differences between infants and older children. In addition, apnea times were shorter because of lower functional residual capacities and higher oxygen consumption, which may lead to faster arterial oxygen desaturation. For this reason, the intubation time must be short [3].

Gupta et al. [1] placed the C-MAC VL size 1 Miller blade and the McGrath MAC size 1 blade in the vallecula and reported a laryngoscopy time of 6.9 s and an intubation time of 27.4 s in the C-MAC group. In the present study, in the group in which the laryngoscope blade was placed in the vallecula, the laryngoscopy time was 5.61 s and the intubation time was 23.16 s. The shorter durations in the present study may be attributable to the older mean age of the participants, the greater experience of the anesthetist performing the intubations, and the fact that all intubations were performed by the same person.

Sinha et al. [8] directly elevated the epiglottis with the C-MAC VL 1 and 0 Miller blades and reported an intubation time, defined as the total procedure time, of 24.5 s. In the present study, the intubation time was 26.60 s in the group in which the epiglottis was directly elevated with the laryngoscope blade. The shorter duration reported by Sinha et al. may be attributable to the higher mean age of their patients and the smaller sample size.

Good glottic visualization does not necessarily result in a shorter intubation time [10]. In a study by Gupta et al. [1] comparing C-MAC VL with McGrath VL, glottic visualization was found to be better with C-MAC VL, but intubation time was found to be similar between the groups. Intubation time was longer in the group in which the epiglottis was directly elevated, although POGO and CL scores and glottic visualization were superior. In the current study, IDS were similar between the groups. However, subjective intubation difficulty was better in the group in which the laryngoscope blade was placed in the vallecula. The difference between IDS and subjective intubation difficulty scores can be explained by the fact that the IDS does not take into account many aspects that may complicate intubation with the VL (e.g., the alignment of the ETT towards the glottis during intubation and blurred images because of evaporation). This concern regarding the use of IDS to measure intubation difficulty was emphasized before [1].

The clinical relevance of the statistically significant differences warrants careful consideration. The absolute difference in POGO score was approximately 4.5 percentage points (95% CI, 0.43–8.55), and the differences in laryngoscopy and intubation times were approximately 1.3 s (95% CI, 0.81–1.87) and 3.4 s (95% CI, 1.83–5.05), respectively. While statistically significant, it remains uncertain whether these differences are clinically meaningful in children with normal airways undergoing elective procedures. In infants with anticipated difficult airways or with limited oxygen reserve, however, even small improvements in glottic visualization or modest reductions in intubation time may carry greater clinical importance; this is a hypothesis that warrants investigation in future studies with higher-risk populations.

Recent evidence has further strengthened the role of video laryngoscopy in neonatal and infant airway management. The 2024 joint ESAIC/BJA guidelines recommend the use of an age-adapted standard-blade video laryngoscope as the first-choice device for tracheal intubation in neonates and infants [11,12]. This recommendation is supported by a recent randomized trial demonstrating substantially higher first-attempt intubation success with video laryngoscopy than with direct laryngoscopy in neonates requiring urgent intubation [13]. Contemporary systematic reviews and meta-analyses have similarly shown improved first-attempt success and glottic visualization with video laryngoscopy, with some evidence of reduced desaturation and airway-related adverse events, although its effect on intubation time remains inconsistent across studies [14,15,16,17,18]. Recent clinical investigations have also highlighted the potential advantages of C-MAC video laryngoscopy in neonates and infants and the influence of operator experience on intubation performance [19,20]. Collectively, these findings reinforce the increasing role of video laryngoscopy in this vulnerable population while emphasizing that optimal blade positioning and the balance between improved glottic visualization and procedural speed remain incompletely defined.

The study had several limitations. First, the intubating anesthesiologist could not be blinded to blade position because the video laryngoscope blade was used in two different positions. However, laryngoscopy and intubation times were well defined and recorded by an independent observer who was unaware of group allocation to reduce potential measurement bias. The primary outcome, the POGO score, was assessed at the end of the study by an independent anesthesiologist who had not participated in the intubation procedures and who evaluated anonymized video recordings without explicit group labels. Nevertheless, because the blade tip and its relationship to the epiglottis could potentially be visible in the recordings, complete blinding of the POGO assessor to the intervention could not be guaranteed, and inadvertent inference of group allocation cannot be excluded, representing a potential source of detection bias. Second, all intubations were performed by a single highly experienced pediatric anesthesiologist, which ensured procedural consistency but may limit the generalizability of the findings to clinicians with different levels of experience. Third, the study included only children with normal airways undergoing elective procedures; therefore, the findings cannot be extrapolated to children with anticipated or known difficult airways or to emergency intubations, which are precisely the populations in whom improved glottic visualization may be most clinically relevant. Fourth, the Cormack–Lehane grade distribution, which predominantly consisted of Grade I views in both groups, suggests a ceiling effect that may have limited the ability to detect clinically meaningful between-group differences. Finally, the Intubation Difficulty Scale may not fully capture the specific nuances of difficulty associated with video laryngoscopy, as it was originally developed for direct laryngoscopy. In addition, the operational definition of intubation time used in this study differed from the prospectively registered definition, as described in Section 2.5; this deviation from the registered outcome definition is acknowledged. These limitations should be considered when interpreting the findings. The clinical relevance of improved glottic visualization is likely to be greatest in children with difficult airways, in whom the margin for error during intubation is narrow and the consequences of failed or prolonged attempts are more serious [11]. However, because the present study included only children with normal airways, the generalizability of the findings to this higher-risk population remains uncertain. Further studies are therefore needed to evaluate the optimal blade-positioning strategy with the C-MAC Miller video laryngoscope in pediatric patients with difficult airways.

## 5. Conclusions

In conclusion, in children younger than 2 years of age undergoing elective surgery with normal airways, direct epiglottic elevation with the C-MAC VL size 1 Miller blade provided superior glottic visualization compared with vallecular placement, whereas vallecular placement was associated with shorter laryngoscopy and intubation times. No intubation-related complications were observed with either technique in this study. The observed differences, although statistically significant, were modest in absolute terms, and their clinical relevance requires further evaluation in higher-risk populations, including children with difficult airways or limited oxygen reserve.

## Figures and Tables

**Figure 1:**
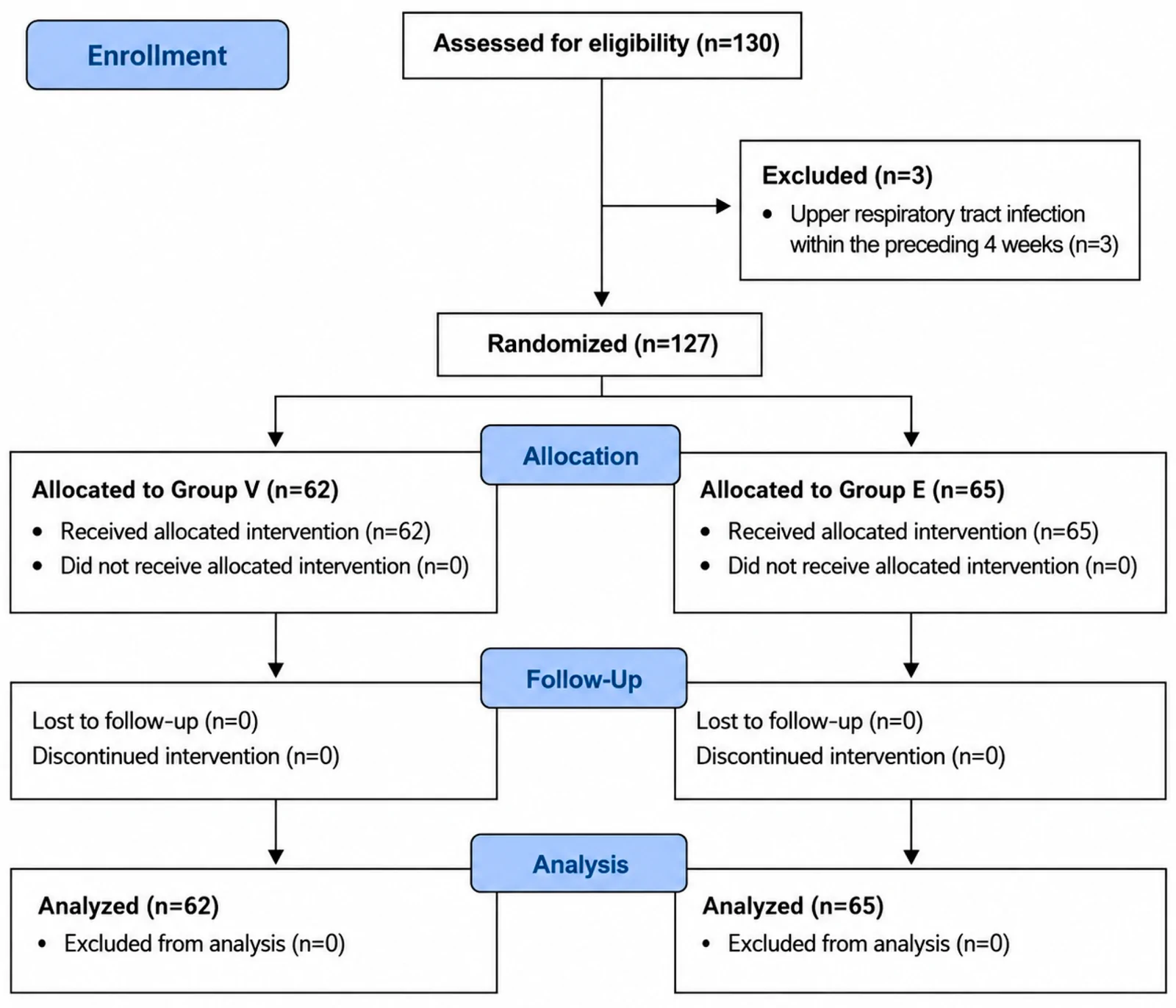
CONSORT flow diagram of participant enrollment, randomization, allocation, follow-up, and analysis.

**Table 1:** Demographic and baseline characteristics of the study groups.

Characteristics	Group V (n = 62)	Group E (n = 65)	*p* Value
Age (months)	13.32 ± 8.26	11.96 ± 7.40	0.346
Body weight (kg)	8.82 ± 3.25	8.84 ± 3.27	0.974
Height (cm)	76.13 ± 14.07	74.37 ± 13.70	0.477
Gender (M/F)	53/9	51/14	0.420
ASA physical status (I/II)	30/32	35/30	0.538

M/F: Male/Female; ASA: American Society of Anesthesiologists physical status classification; *p* < 0.05: statistically significant.

**Table 2:** Intubation characteristics.

Outcomes	Group V (n = 62)	Group E (n = 65)	Effect Estimate (95% CI)	*p* Value
POGO score (%), median (IQR)	95 (88–100)	96 (92–100)	Median difference 2.0 (0.5 to 4.0)	0.032 *
POGO score (%), mean ± SD	90.31 ± 15.83	94.80 ± 5.32	MD 4.49 (0.43 to 8.55)	0.032 *
Cormack–Lehane score (I/II/III/IV)	54/6/2/0	64/1/0/0	—	0.042 *
Laryngoscopy time (s)	5.61 ± 1.15	6.95 ± 1.80	MD 1.34 (0.81 to 1.87)	0.001 *
Intubation time (s)	23.16 ± 4.44	26.60 ± 4.73	MD 3.44 (1.83 to 5.05)	0.001 *
VAS (0–10)	9.48 ± 1.09	9.02 ± 1.09	MD −0.46 (−0.84 to −0.08)	0.018 *
IDS	0.59 ± 1.24	0.49 ± 0.75	—	0.567
Intubation attempt (I/II)	61/1	65/0	—	0.488

Continuous variables are presented as mean ± SD or as median (IQR). The between-group effect estimate for the primary outcome (POGO score) is presented as the Hodges–Lehmann median difference (Group E − Group V) with its 95% confidence interval, with the mean difference (MD) from the parametric sensitivity analysis also reported. MD, mean difference; POGO, Percentage of Glottic Opening; VAS, Visual Analogue Scale; IDS, Intubation Difficulty Score; CI, confidence interval; IQR, interquartile range. * *p* < 0.05 indicates statistical significance.

**Table 3:** The number of optimization maneuvers.

Optimization Maneuvers	Group V (n = 62)	Group E (n = 65)	*p* Value
BURP	9	11	0.898
Rotation	0	0	1.00
Lifting	11	11	1.00

BURP: Backward, upward and rightward pressure.

## Data Availability

The data presented in this study are available on request from the corresponding author. The data are not publicly available due to privacy and ethical restrictions.

## Supplementary Materials

The following supporting information can be downloaded at: https://www.mdpi.com/article/10.3390/jcm15197483/s1, CONSORT 2025 checklist of information to include when reporting a randomized trial [21].
